# The Spatiotemporal Control of Osteoblast Cell Growth, Behavior, and Function Dictated by Nanostructured Stainless Steel Artificial Microenvironments

**DOI:** 10.1186/s11671-016-1810-1

**Published:** 2017-02-06

**Authors:** Udesh Dhawan, Hsu-An Pan, Meng-Je Shie, Ying Hao Chu, Guewha S. Huang, Po-Chun Chen, Wen Liang Chen

**Affiliations:** 1 Department of Materials Science and Engineering, National Chiao Tung University, 1001 University Road, Hsinchu, Taiwan, ROC; 2 Institute of Materials Science and Engineering, National Taipei University of Technology, Section 3, Zhongxiao E Road, Taipei City, 106 Taiwan, ROC; 3 Department of Biological Science and Technology, National Chiao Tung University, 1001 University Road, Hsinchu, 300 Taiwan, ROC

**Keywords:** Nanotopography, Osteoblast, Nanoporous, Stainless steel, Extracellular matrix, Artificial microenvironment

## Abstract

The successful application of a nanostructured biomaterial as an implant is strongly determined by the nanotopography size triggering the ideal cell response. Here, nanoporous topography on 304L stainless steel substrates was engineered to identify the nanotopography size causing a transition in the cellular characteristics, and accordingly, the design of nanostructured stainless steel surface as orthopedic implants is proposed. A variety of nanopore diameters ranging from 100 to 220 nm were fabricated by one-step electrolysis process and collectively referred to as artificial microenvironments. Control over the nanopore diameter was achieved by varying bias voltage. MG63 osteoblasts were cultured on the nanoporous surfaces for different days. Immunofluorescence (IF) and scanning electron microscopy (SEM) were performed to compare the modulation in cell morphologies and characteristics. Osteoblasts displayed differential growth parameters and distinct transition in cell behavior after nanopore reached a certain diameter. Nanopores with 100-nm diameter promoted cell growth, focal adhesions, cell area, viability, vinculin-stained area, calcium mineralization, and alkaline phosphatase activity. The ability of these nanoporous substrates to differentially modulate the cell behavior and assist in identifying the transition step will be beneficial to biomedical engineers to develop superior implant geometries, triggering an ideal cell response at the cell-nanotopography interface.

## Background

Nanotopography modulates cellular behavior [1–3]. A plethora of nanotopographies such as nanodots [4, 5], nanoislands [2, 6], nanoridges [7], nanotubes [8, 9], and nanoconcave [10] have been known to dictate the cellular behavior in the form of cell morphology, cell migration, cell viability, and cell physiology [11]. A diversity of materials such as tantalum oxide [12], titanium dioxide [13], and mica [14] have been exploited to develop nanosurfaces with nanotopographies and nanosurfaces which the body may identify as “self,” thereby providing an ideal environment for the cells to attach and grow. In the past, studies on fibroblasts [15], cardiomyocytes [16], osteoblasts [17, 18], and cancerous cell lines such as C33A, TOV-112D, and TOV-21G [19] have shown how the nanotopography can guide the cellular behavior in the form of cell area, focal adhesions, and microfilament bundles. Cells have been seen to respond favorably to a variety of nanotopographies between the size ranges of 20 to 100 nm [4]. Significant reduction in cell viability in addition to an elongated or an apoptotic morphology accompanied with scarce focal adhesions has been observed for nanotopographies above 100 nm [20]. Modulation in the form of Integrins further elucidates how cellular behavior and ultimately the cell fate can be dictated in vitro by nanotopographies in different size range, at the genetic level [21].

Physical properties of the nanosurface such as roughness [21], shape [19], and stiffness [22] modulate the in vitro cell behavior in a way which is similar to extracellular matrix (ECM) in vivo. Studies have shown that the implant topography affects the amount of bone deposited next to the implant [23]. Thus, nanotopography is considered an important factor in dictating cell behavior. In addition, nanotopography has also been seen to modulate the bone mineralization [24]. Thus, it is believable that the size, shape, and the arrangement of nanotopographic features modulate the cellular response. Among all the materials exploited for applications as implants, metallic biomaterials such as stainless steel tend to be the first choice primarily due to their high strength and fracture and corrosion resistance for an extended period of time [25–27]. However, the number of studies utilizing stainless steel as a substrate to engineer nanotopographies for improved cell attachment, growth, and function are scarce. Engineering nanotopographies to elucidate the parameters causing a transition in the cell behavior may help the biomedical engineers to understand the modulation in the cellular behavior which may act as a cornerstone in designing implants with surface geometries which cause the cells to respond to them favorably.

The present study is based on the hypothesis that nanoporous topographies of different diameters may cause a transition in the cellular behavior and may guide osteoblast cell attachment, growth, function, and ultimately, the fate. Nanoporous topography ranging from 100 to 220 nm was fabricated by a simple electrolysis method in perchloric acid and ethylene-glycol monobutylether by varying the voltages. MG63 osteoblasts were used as a model for this study. In particular, the aim of this study was to engineer a nanotopography which highlights the transition step in the modulation of cell characteristics in response to the nanotopography. Finally, the optimized nanosurface geometry for application as orthopedic implants is proposed. The findings of this study may find applications in the fields of nanobiotechnology, biomedical engineering, tissue engineering, and cancer research.

## Methods

### Fabrication of Nanoporous Stainless Steel Substrates

The 304L stainless steel was used for the purpose of engineering nanoporous surfaces. The dimensions of surfaces were 25mm × 25mm × 2mm and provided with a thread for the electrical contact. The composition of stainless steel substrates was (wt. %) chromium (Cr) 18.68%, nickel (Ni) 10.14%, manganese (Mn) 1.72, molybdenum (Mo) 0.35, copper (Cu) 0.15%, nitrogen (N) 0.072%, carbon (C) 0.018, and Fe balanced. First, the surfaces were mechanically polished with abrasive papers (grades 500, 1200, 2400, and 4000) followed by diamond pastes of decreasing grades (3, 1, and 0.25 m). Samples were ultra-sonicated in acetone, ethanol, and distilled water for 10 min each. Next, the electrolysis was carried out in an electrolytic bath. For the duration of electrolysis, the temperature of the bath was maintained between −5 and 15 °C. Forty microliters of perchloric acid and 760 mL of ethylene-glycol monobutylether were mixed together and used as electrolytes. Perchloric acid was used to obtain a low PH to promote ionization of metallic atoms into cations instead of oxides. Ethylene-glycol monobutylether maintains the viscosity of the electrolyte. Voltage used for carrying out electrolysis was varied to control the diameter of nanopores. Electrolysis was carried out at 45, 60, 70, and 75 V to fabricate nanopores of 100, 180, 200, and 220 nm diameter. Nanopores of less than 100 nm could not be fabricated due to limitation of the fabrication set up resulting in inhomogeneity in the diameter of nanodots. The dimensions and homogeneity of nanopores were analyzed with JEOL JSM-6500 TFE SEM. The diameters of the nanopores were well controlled and highly defined.

### Cell Culture

MG63, osteoblast-like cells, originally isolated from human osteosarcoma, were seeded on the nanoporous substrates and cultured in Eagles’ minimum essential medium supplemented with 2 mM l-glutamine and Earle’s BSS containing 1.5 g/l sodium bicarbonate, 0.1 mM non-essential amino acids, 1.0 mM sodium pyruvate, and 10% fetal bovine serum. Cell cultures were incubated in a humidified incubator with 5% CO_2_ at 37 °C. Cell culturing was performed in a class-10 clean room to ensure elimination of any possible contamination.

### Morphological Analysis by Scanning Electron Microscopy

MG63 osteoblasts were seeded on the control (flat) and nanosurfaces with different pore diameter, harvested after days 1 and 3, and the transition in the cell morphology was observed. The culture medium was removed, and the wells were washed twice with Dulbecco’s phosphate-buffered saline (DPBS) followed by fixation in 1.25% glutaraldehyde (Electron Microscopy Sciences, USA) (in PBS) for 20 min at room temperature. Post-fixation, the cells were covered with 1% osmium tetroxide (Electron Microscopy Sciences, USA) solution for 30 min. Samples were washed thrice with PBS and finally immersed in 40% alcohol overnight at 4 °C. Next day, sequential dehydration was performed with a series of alcohol concentrations (10-min incubation in 50, 60, 70, 80, 90, 95, and 100%). The samples were then sputter-coated with platinum, and cell morphology was examined with JEOL JSM-6500 TFE SEM at an accelerating voltage of 8 or 10 kiloelectron volts (KeV).

### Immunostaining

MG63 osteoblasts were seeded on control (flat) and nanosurfaces with different pore diameter, harvested after days 1 and 3, and the transition in morphology, cell area, focal adhesions, and vinculin-stained area was analyzed. The culture medium was removed, and the cells were washed twice with Dulbecco’s phosphate-buffered saline (DPBS) followed by fixation with 4% paraformaldehyde for 15 min at room temperature. Samples were then washed thrice with PBS. The samples were then permeabilized in 0.1% Triton X-100 for 10 min followed by three washes in PBS. Blocking was performed in 2% BSA (Thermo Fisher Scientific, USA), prepared in PBS, for 1 h at room temperature on the orbital shaker, and followed by overnight incubation at 4 °C. Next day, samples were washed thrice with PBS followed by incubation with anti-vinculin antibody (Bioss USA), diluted in 2% BSA, for overnight at 4 °C. Next day, the samples were washed thrice with PBS and then incubated with goat anti-rabbit FITC-conjugated secondary antibody (Jackson Immunoresearch, USA) and Alexa Fluor 594 for cytoskeletal staining for 1 h at room temperature. Finally, the samples were washed thrice with PBS, mounted on glass slides, and examined using a Leica TCS SP2 confocal microscope. The number of focal adhesions was calculated by counting the green spots (vinculin stained), and the cell area was analyzed using ImageJ software. For the statistical analysis of the number of focal adhesions, the number of vinculin stained spots were counted, normalized against control (flat) surfaces and expressed as the number of focal adhesions/cell. Vinculin area was also analyzed and expressed as vinculin stained area/cell (μm^2^). Similar methodology was adopted for statistical analysis of cell cytoskeleton and expressed as cell cytoskeleton/μm^2^. All washes were performed in 1× PBS for 5 min each.

### Measurement of Cell Growth and Cell Spreading Area

Cells were seeded on control (flat) and nanosurfaces with different pore diameter, harvested after days 1 and 3. The adhered cells were fixed in 1.25% glutaraldehyde (in PBS) for 20 min at room temperature, followed by three washes with PBS. The following steps for sample preparations are same as mentioned in “Immunostaining” section. Finally, SEM was used to analyze which nanoporous surfaces promoted a greater cell growth. The number of cells were counted using ImageJ software. For statistical analysis, six different substrate fields were measured per sample, and three separate samples were analyzed. The mean was then normalized against the control (flat) surfaces and expressed as cell number/mm^2^. To analyze which nanoporous surface promoted a greater cell spreading area, the area was measured using ImageJ, normalized against cell spreading area on flat surfaces, and expressed as cell spreading area (%).

### Measurement of Cell Viability

The procedure to calculate the cell viability was followed as described elsewhere [21]. Briefly, cells were seeded on control (flat) and nanosurfaces with different pore diameter, harvested after days 1 and 3. Samples were washed thrice with DPBS, fixed in 4% paraformaldehyde, and stained with 4′,6-diamidino-2-phenylindole (DAPI) (Thermo Fisher Scientific, USA). Samples were incubated for 20 min, in dark, followed by mounting on a glass slide and imaging with Leica TCS SP2 confocal microscope.

### Alizarin Red S Staining

Cells were seeded at a density of 7 × 10^3^ cells/cm^2^ on control (flat) and nanosurfaces with different pore diameter. Cells were harvested after days 7, 10, and 14, and alizarin red S staining was then performed to analyze the extent of calcium mineralization. Samples were fixed in 4% paraformaldehyde, prepared in PBS for 10 min at room temperature, followed by soaking in 2% alizarin red S, prepared in DI water (PH adjusted to 4.2), at 37 °C for 20 min. Samples were then washed with DI water to remove any excess stain. The samples were observed under a microscope. For statistical analysis, 50 cells were randomly picked and the area of stain per cell on each nanoporous surface, relative to area of stain per cell on flat surfaces, was calculated.

### Alkaline Phosphatase Assay

Alkaline phosphatase activity was determined with alkaline phosphatase assay kit following the manufacturer’s instructions (Abcam, Taiwan). Briefly, cells were seeded on control (flat) and nanosurfaces with different pore diameter. Alkaline phosphatase activity was analyzed after days 7, 10, and 14. First, the cells were lysed in 1× lysis buffer (Tris-Cl, PH 7.4, NaCl, EDTA, Triton X-100, PMSF, proteinase inhibitor cocktail tablet, and water), scraped, and centrifuged at 12,000*g* for 2 min at 4 °C. Supernatants were removed and then transferred to new Eppendorf tubes. UV/OD was used to define the protein concentrations. Twenty microliters of sample buffer and 100 μl of pNPP substrate were mixed and incubated in dark for 30 min. Measurements were taken at 405 nm on a multi-well plate reader.

### Statistical Analysis

Results were expressed as the mean ± standard deviation of *n* experiments (*n* ≥ 3). For the statistical analysis, one-way analysis of variance followed by Tukey’s post-test (SPSS 13.0 software, Chicago, SA) was used, and the level of significance was set at *P* < 0.05. Highly significant values were expressed as ** with the level of significance <0.01.

## Results

### Fabrication of Nanoporous Surfaces

**Fig. 1:**
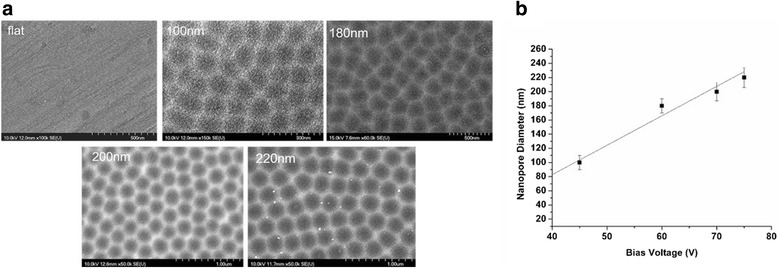
Fabrication of nanoporous stainless steel artificial microenvironments. **a** Scanning electron microscopy of nanoporous surfaces on stainless steel substrates. Highly defined nanopore diameters between 100 and 220 nm were fabricated. *Scale bar* for flat = 500 nm, 100 = 300 nm, 180 = 500 nm, 200, and 220 = 1 μm. **b** Graphical representation depicting a linear relationship between anodization voltage and nanopore diameter

Nanoporous surfaces with a pore diameter of 98 ± 6, 180 ± 8, 197 ± 10, and 218 ± 12 were fabricated as described in “Fabrication of nanoporous surfaces” section. After electrolysis, the pore diameter was analyzed with the help of scanning electron microscopy (SEM) (Fig. 1a). Four nanoporous surfaces with different nanopore diameters were fabricated. The diameter of the nanopores was directly proportional to the bias voltage supplied during the fabrication procedure (Fig. 1b). For control surfaces, polished stainless steel substrates were used. The diameter of the nanoporous surfaces was well controlled and highly defined.

### Nanoporous Surfaces Modulated the Osteoblast Cell Morphology, Spreading Area, and Growth

**Fig. 2:**
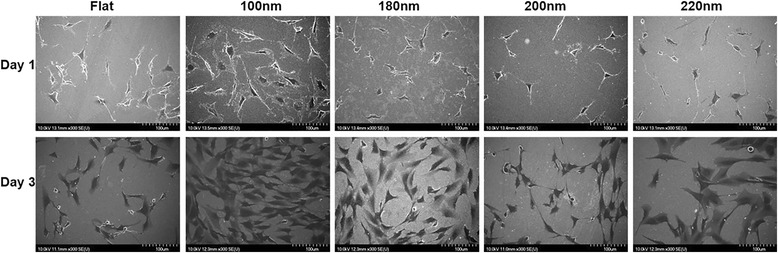
Scanning electron microscopy of cells cultured on various nanoporous stainless steel artificial microenvironments. Cells were harvested after days 1 and 3. Cells on 100 nm displayed a well-extended morphology, complemented by a large cell area as compared to a shrunken morphology on 200 and 220 nm. After day 1, a substantial amount of lamellipodia were observed in cells on 100-nm nanoporous surfaces in contrast to scarce or none on 200- and 220-nm nanoporous surfaces. After day 3, a greater number of cells were observed on 100 nm than on any other nanoporous surface, indicating that 100 nm provided an ideal environment for cell attachment and growth. Transition in cell characteristics was observed as the nanopore size became more than 100 nm. All images were taken at 300×. *Scale bars* = 100 μm

Osteoblast cells were cultured on different nanoporous surfaces having different pore diameter, for days 1 and 3, and the modulation in cell morphology was observed with the SEM. On day 1, the transition in cell morphology with the variation of nanopore diameter was identified (Fig. 2). Cells displayed a well-extended morphology with a plethora of lamellipodia on the flat (control) and 100-nm nanoporous surfaces which transitioned to an abnormal/shrunken morphology, accompanied with scarce lamellipodia on the 200- and 220-nm nanopore surfaces (Fig. 2). On day 3, cells on 100 nm had comparatively more extended morphology than on any other nanosurface (Fig. 2). However, no substantial difference was seen in the morphology as the nanopore size became more than 100 nm.

**Fig. 3:**
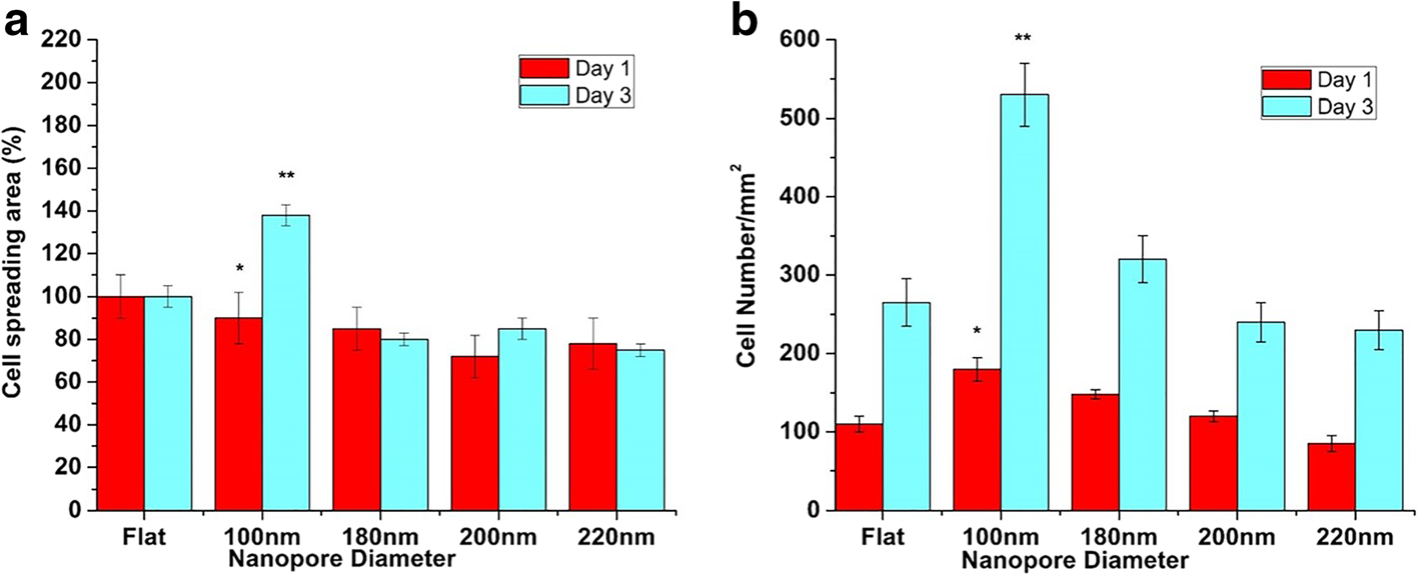
Statistical analysis of cell spreading area (%) and cell number (/mm^2^) on different nanoporous stainless steel artificial microenvironments. **a** After day 1, no significant difference in the cell spreading area was observed in cells on control and 100-nm nanoporous surfaces. After day 3, cell spreading area percentage maximized at 100 nm. Consistent decrement in cell spreading area percentage was observed as the nanopore diameter became more than 100 nm. One hundred-nanometer nanoporous surfaces acted as the transition-inducing factor for cell spreading area. **b** After day 1, cell number maximized at 100-nm nanoporous surfaces. Consistent decrement in cell number was observed as the nanopore diameter became more than 100 nm. One hundred-nanometer nanopore diameter acted as the transition-inducing factor for cell number. Statistically significant data is represented by * having *p* < 0.05; highly significant values are represented with ** having *p* < 0.01

Transition in the cell spreading area corresponding to the nanopore diameter was identified. Cells on 100-nm nanoporous surfaces displayed more cell spreading area as compared to the cells on 200- and 220-nm nanoporous surfaces (Fig. 3a). The value of cell area on 100 nm nanoporous substrates was highly significant when compared to other nanoporous surfaces. A greater number of cells were observed on 100-nm nanoporous surfaces as compared to 200- and 200-nm nanoporous surfaces, indicating a greater cell growth on 100-nm nanoporous surfaces after days 1 and 3 (Fig. 3b). The amount of cell number on 100-nm nanoporous surfaces was also found to be highly significant (*p* < 0.01).

In summary, nanoporous surfaces induced a transition in the cell characteristics. The 100-nm nanoporous allowed the cells to have a well-extended morphology with a plethora of lamellipodia (Fig. 2), a greater cell spreading area (Fig. 3a) and promoted a greater cell growth (Fig. 3b).

### Nanoporous Surfaces Modulated the Osteoblast Cell Cytoskeleton, Focal Adhesions, and Cell Adhesion

**Fig. 4:**
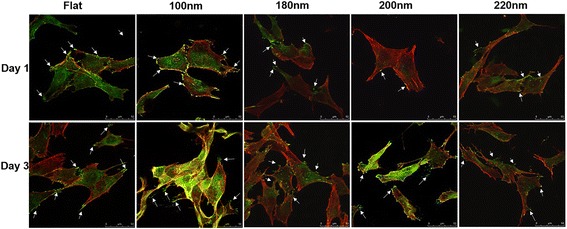
Immunofluorescence staining of cells cultured on various nanoporous stainless steel artificial microenvironments. Cells were seeded on different artificial microenvironments and harvested after days 1 or 3. Cytoskeletal arrangement was examined using phalloidin, and vinculin localization was examined using anti-vinculin antibodies (*green dots* or *green-stained area*). After day 1, cells displayed an extended morphology on 100-nm nanoporous surfaces, which transitioned to an abnormal elongated or shrunken morphology on 220-nm nanoporous surfaces. A plethora of focal adhesions were seen in cells on flat, 100-nm nanoporous surfaces as compared to 200- and 220-nm nanoporous surfaces. *Arrows* represent the green spots, i.e., focal adhesions. A similar trend in cell morphology and the number of focal adhesions was seen after day 3. All images were taken at 400×. *Scale bars* = 50 μm

Transition in the number of focal adhesions from cells on to the various nanoporous surfaces was observed and identified by staining vinculin. Cells on 100-nm nanoporous surfaces stained vinculin at the point of contacts between cells and the surface (focal adhesions) as well as in the cytoplasm (Fig. 4). However, cells on 200- and 220-nm attenuated vinculin expression and thus stained vinculin primarily in the cytoplasm and displayed scarce focal adhesions (Fig. 4).

**Fig. 5:**
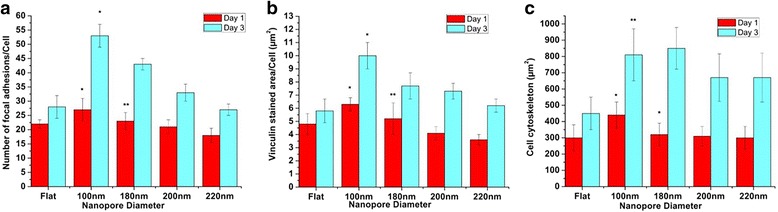
Statistical analysis of cell cytoskeletal area, number of focal adhesions, and vinculin-stained area on different nanoporous stainless steel artificial microenvironments. **a** Cells on 100 nm displayed the maximum number of focal adhesions from cells onto the nanoporous surfaces on both the days. One hundred nanometers acted as the factor in causing the transition in the number of focal adhesions. **b** Maximum vinculin-stained area was seen in cells on 100-nm nanoporous surfaces for both the days. A consistent decrement in the vinculin-stained area was observed with an increment in the nanopore diameter. One hundred-nanometer nanopore diameter was marked as the transition step for vinculin expression. **c** After day 1, maximum cell area was displayed by cells on 100 nm which decreased consistently with an increase in the nanopore diameter. After day 3, cells on 100 nm still displayed the maximum cell area. Least cell area was seen in cells on 200- and 220-nm nanoporous surfaces after both the days. One hundred-nanometer nanopore diameter was marked as the transition-inducing factor for cell area. * represents statistically significant values with *p* < 0.05, while ** represents highly significant values with *p* < 0.01

The number of focal adhesions per cell was counted, and transition step in increment or decrement with respect to control (flat) surfaces was identified and then plotted against the incubation time. After day 1, the number of focal adhesion was highest on cells grown on 100-nm nanoporous surfaces as compared to any other nanosurface (Fig. 5a). The values of focal adhesions on different days were found to be significant (*p* < 0.05). A consistent decrement was observed in the number of focal adhesion on cells cultured on 200- and 220-nm nanoporous surfaces (Fig. 5a). A similar trend yet a significantly greater difference was observed after day 3 (Fig. 5a). Cells on 100-nm nanoporous surfaces had twice the number of focal adhesions than the cells on flat surfaces. In addition, the focal adhesion number decreased consistently with the increase of nanopore diameter. The number of focal adhesions on 200-nm nanoporous surface reached the control level (Fig. 5a). The vinculin-stained area per μm^2^ was also analyzed, and a trend consistent to the number of focal adhesions was observed (Fig. 5b). Vinculin-stained area was maximized on 100-nm nanoporous surface. Nanopores with 100-nm diameter significantly promoted the expression of vinculin and the number of focal adhesions as compared to 200 and 220 nm (Fig. 5b).

A tight cytoskeleton representing a well-extended morphology was observed in cells cultured on 100-nm nanoporous surfaces but lost order in the cells cultured on 200- and 200-nm nanoporous surfaces after days 1 and 3 (Figs. 4 and 5c). In summary, a well-organized cell cytoskeleton with a plethora of focal adhesions was observed in cells cultured on 100-nm nanoporous surfaces. The 200- and 220-nm nanopore diameter retarded the cytoskeletal organization displaying an elongated or a shrunken morphology. Thus, the nanopore diameter played a vital role in the transition of vinculin expression, focal adhesions, and cell cytoskeleton organization.

### Nanoporous Surfaces Modulated the Cell Viability

**Fig. 6:**
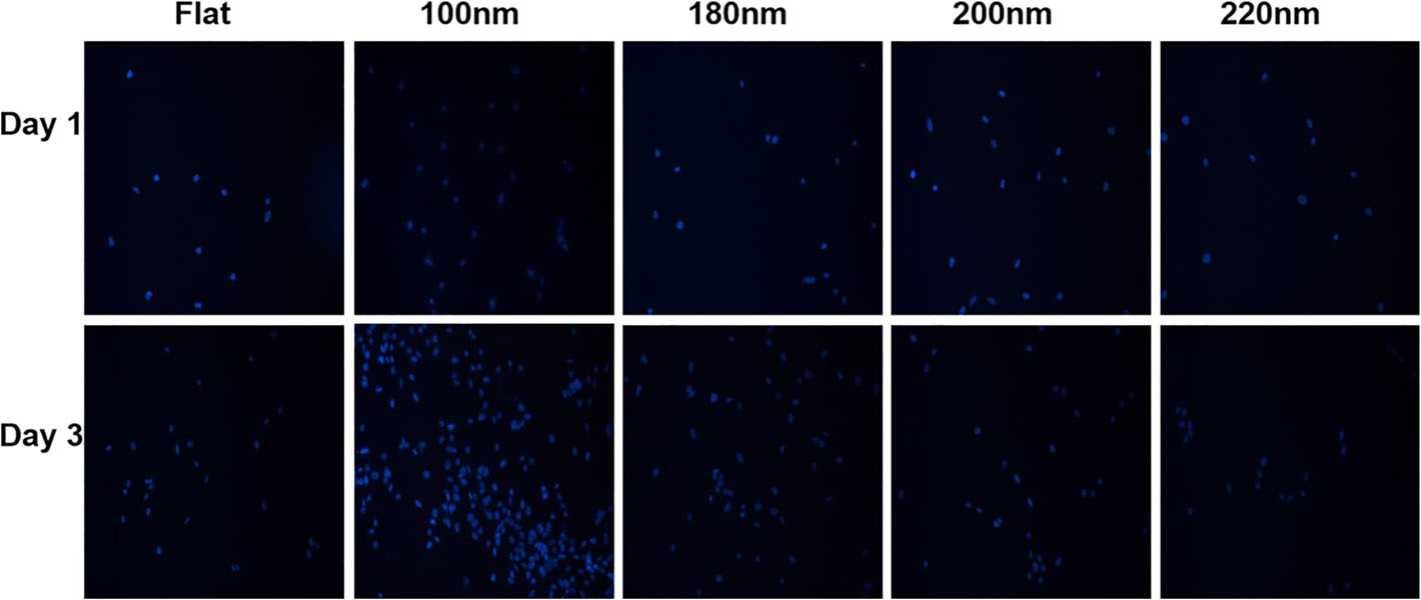
Immunofluorescent labeling of cell nucleus on different nanoporous artificial microenvironments. Cell nucleus was labeled blue with DAPI to analyze the number of viable cells after days 1 and 3. For both the days, the maximum number of viable cells were seen on 100-nm nanoporous surfaces. One hundred-nanometer nanopore diameter was marked as the transition step in the modulation of cell viability by the nanoporous surfaces

Cells were seeded on different nanoporous surfaces, and the cell viability was evaluated after days 1 and 3. After day 1, the number of viable cells on 100-nm nanoporous surfaces was considerably more than on any other nanosurface. However, a consistent decrement was observed in the cell viability on 200- and 220-nm nanoporous surfaces. After day 3, the number of viable cells on 100-nm nanoporous surfaces was significantly higher than on any other nanoporous surface (Fig. 6). However, a consistent decrement was observed in cells cultured on 200- and 220-nm nanoporous surface. Thus, the nanopore diameter was responsible for causing the transition in the number of viable cells on different nanosurfaces (Fig. 6). In summary, 200- and 220-nm nanoporous surfaces caused cell death which can be attributed to the greater diameter of nanopores.

### Nanoporous Surfaces Modulated the Osteoblast Mineralization

**Fig. 7:**
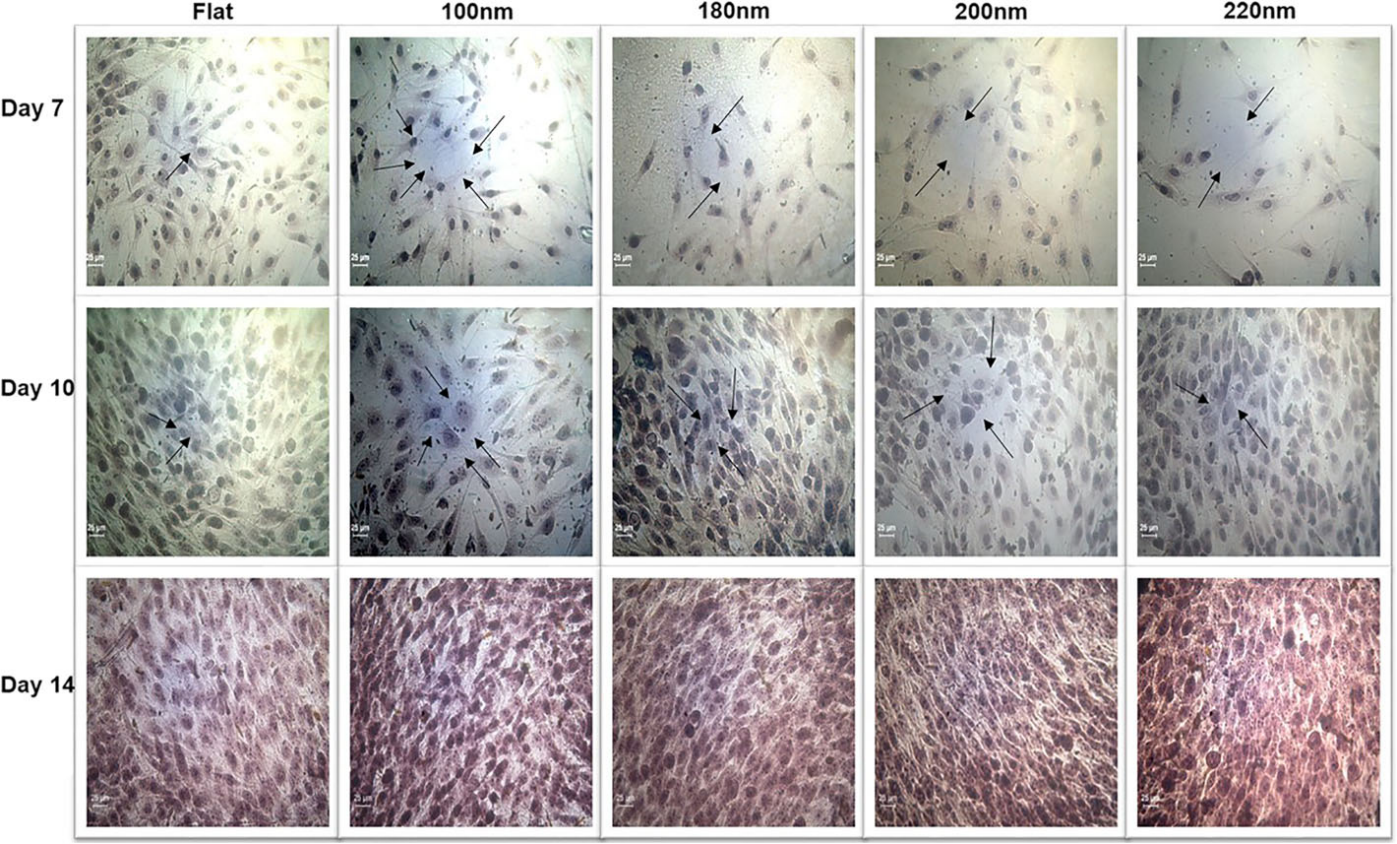
Alizarin red S staining to visualize mineral calcium deposition. Cells were cultured on different nanoporous artificial microenvironments for 7, 10, and 14 days, and alizarin red S staining was then performed. Maximum staining intensity signifying maximum calcium mineral deposition was observed in cells on 100-nm nanoporous surfaces. *Arrows* point to the purple-stained sections on nanosurfaces. Mineral deposition maximized on day 14 in cells on 100-nm nanoporous surfaces. *Scale bar* represents 25 μm

**Fig. 8:**
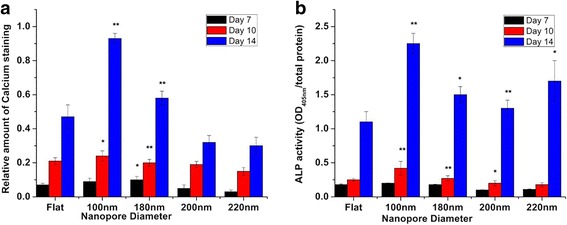
Statistical analysis of alizarin red S staining and ALP assay in MG63 osteoblasts cultured over different nanoporous artificial microenvironments. **a** The extent of calcium mineralization on various nanosurfaces over different days calculated from the alizarin red S staining. Maximum mineralization was seen in cells on 100-nm nanoporous surfaces for days 10 and 14. A consistent decrement in mineralization was observed as the nanopore diameter became greater than 100 nm. One hundred-nanometer nanopore diameter marked as the transition step in regulating the calcium mineralization. **b** ALP assay of cells over different days. Maximum ALP activity was observed in cells on 100-nm nanoporous surfaces. One hundred-nanometer nanopore diameter was recognized as the transition step for regulating the ALP activity in the cells. * represents statistically significant values with *p* < 0.05, ** represents highly significant values with *p* < 0.01

The extent of mineralization is an important factor to understand the osteoblast function. Alizarin red S staining was used to study the extent of mineralization in osteoblasts cultured on flat and nanoporous surfaces. Cells were harvested after days 7, 10, and 14, and quantitative measurement of mineralization was performed. The calcium deposits were stained purple. Nanopore diameter caused the transition in the extent of mineralization by the osteoblasts. After day 7, the amount of calcium deposits were higher in cells cultured on 100-nm nanoporous surfaces than on 200- and 220-nm nanoporous surfaces (Fig. 7). After day 10, mineralization extent was found to be highest on 100-nm nanoporous surfaces (Fig. 7). However, a consistent decrement was observed in cells cultured on 200- and 220-nm nanoporous surfaces. Similar trend was seen after day 14 (Fig. 7). The highest mineralization amount was observed in cells seeded on 100-nm nanoporous surfaces. Thus, the nanopore diameter was associated with causing the transition in the mineralization by the osteoblasts (Figs. 7 and 8a).

### Nanoporous Surfaces Modulated the Alkaline Phosphatase Activity

A high concentration of phosphate is created at the mineralization front. Calorimetric alkaline phosphatase (ALP) assay was used to determine the modulation in the level of alkaline phosphatase secretion in response to nanoporous surfaces. Cells were cultured on different nanoporous surfaces for days 7, 10, and 14, and ALP activity was analyzed. ALP activity was measure at OD_405nm_. Nanopore diameter caused the transition in the ALP activity. After day 7, detectable quantity of ALP was observed on all nanoporous surfaces. However, significantly higher ALP activity was observed in cells cultured on 100 nm nanoporous surfaces than on any other nanoporous surface (Fig. 8b). After day 10, ALP activity was still the highest in cells cultured on 100-nm nanoporous surfaces. However, a consistent decrease was observed in cells cultured on 200- and 220-nm nanoporous surfaces (Fig. 8b). After day 14, ALP activity on 100-nm nanoporous surfaces was 80, 120% higher than on the flat surfaces. The highest ALP activity was observed on 100-nm nanoporous surfaces (Fig. 8b). Thus, the nanopore diameter played a vital role in the transition of ALP activity in the osteoblasts.

## Discussion

Cell characteristics such as cell morphology, area, motility, and fate are closely related to the physical attributes of their microenvironment. Engineering a topography in the nanorealm should be the primary task for the aim of developing a biocompatible nanosurface. Multiple attempts have been made in the past by our research group to explore how different kinds of cells respond to different shapes and sizes of nanotopographies [4, 11]. We have shown that while cells respond favorably to tantalum oxide nanodots with size less than 50 nm, they display distinct characteristics of apoptosis such as an apoptotic morphology, scarce focal adhesions, and reduced cell area when the nanodot size reaches 100 nm [5, 18, 20]. Thus, finding a suitable nanotopography shape and size or a particular application is rather hard. However, the choice of material on which nanotopographies are engineered varies with its application. Stainless steel has been the primary choice of material for constructing orthopedic, dental, or surgical implants due to the favorable combination of strength, fabrication properties, and minimal in vivo cytotoxicity [28]. This is particularly beneficial when compared to other materials such as alumina and zirconia which display undesirable surface properties, uncontrollable degradation, and utilizing uncommon techniques for fabrication [29]. Another important aspect which may help the biomedical engineers to develop more biocompatible geometries for applications as implants is identifying the transition step in the modulation of cell characteristics and behavior.

In this study, we used stainless steel as a substrate and engineered nanoporous topographies from 100 to 220 nm (Fig. 1a) to understand how variation in the size of nanotopographies can guide the cellular behavior and thereby providing an optimized nanosurface geometry for application as orthopedic implants. The morphology analysis with SEM revealed that the 100-nm nanoporous surfaces provided an ideal environment for the cells to attach and grow (Fig. 2). Cells’ spreading area (Fig. 3a) and density (Fig. 3b) were found to decrease with an increment in the size on the nanopore diameter. In addition, the number of lamellipodia seen on cells cultured on 100-nm nanoporous surfaces were more as compared to on any other nanopore diameter (Fig. 2). These results are in firm agreement with our previous studies on fibroblasts [21]. Another important conclusion that can be drawn from these results is that the 100 nm promoted cell growth while 200- and 220-nm nanoporous surfaces affected the cell growth negatively (Fig. 3b). These results were further confirmed when cellular cytoskeleton and focal adhesions (vinculin) were stained. Cellular cytoskeleton was ordered (Fig. 4) with cells displaying a plethora of focal adhesions (Fig. 5a, b) on to the nanosurface. Focal adhesions are important for cell attachment, and thus, growth [30]. It is therefore a reasonable conclusion that 100-nm nanoporous surfaces provided a friendly environment for the cells to attach and grow which led to their ordered, well-extended cytoskeleton (Fig. 5c) and morphology while 200- and 200-nm nanoporous surfaces caused cell death due to scarce focal adhesions (Fig. 5a). In addition, these results are consistent with our previous studies on nanodots where cells on 200-nm nanodots displayed an apoptotic morphology with very few focal adhesions [20]. Also, our studies on fibroblasts have proved that nanotopographies with size 200 or more do not trigger the cells to activate integrins, necessary for focal adhesion formation [21]. Consequently, cells on 100-nm nanoporous surfaces displayed maximum vinculin-stained area than on any other nanosurface.

For applications as implants, culturing cells over the material for an extended period of time is very crucial. Previous studies have shown that when the topography size became more than 50 nm, reduction in mineralization was observed [18]. While in the previous study nanodots were investigated as an appropriate material, [31–33] in this study, nanoporous topography was investigated. Thus, a small variation in the size of the nanotopography suitable for orthopedic implant application is reasonable. The results of alizarin red staining (Figs. 7 and 8a) and the measurement of alkaline phosphatase activity (Fig. 8b) were not only consistent with our previous studies [18] but also with the other results of this study where cells displayed enhanced cell characteristics on 100 nm than on any other nanopore diameter. At the moment, the only possible explanation of why some nanosurface shapes and sizes trigger the cells to respond in a way as if they were growing in vivo can be the partially resemblance of the in vivo ECM components.

While the focus of this study was stainless steel, other materials such as titanium dioxide [34], tantalum oxide [18], nickel [35], nickel-titanium alloys [36], ceramics [37], polystyrene [38], glass [39], and plastic [40] have also been exploited to study the osteoblast behavior. However, the osteoblast behavior as a function of nanopore diameter has never been studied before. Moreover, the effect of nanopore diameter in acting as a transition step to modulate the cell behavior has also never been identified. Of course, other nanosurface factors such as surface roughness and nanopore depth may also affect the cell behavior along with other possible application of these nanoporous surfaces as cardiac implants, which will remain to be the subject of the future studies. Importantly, just as any biomaterial, stainless steel too possesses certain disadvantages such as mild corrosion and mismatch of physical properties with the soft tissue adjacent to the bone. However, these difficulties can be partially overcome by coating the substrates with oxides such as those tantalum. Therefore, overcoming these difficulties to generate a highly biocompatible biomaterial will remain to be the focus of upcoming studies. Collectively, the results of this study demonstrate the role of nanoporous topography in modulating the osteoblast cell behavior, in addition to identifying the nanopore diameter causing the transition in the cell behavior. Specifically, the results of this study can be utilized in the design of orthopedic implant nanosurface. In general, applications in the fields of biomedical, tissue engineering, and cancer research are expected.

## Conclusions

In the present study, the role of nanoporous stainless steel geometries in causing transition in osteoblast cell characteristics such as cell morphology, spreading area, growth, viability, focal adhesions, and function was investigated. Transition step causing the modulation in the osteoblast cell behavior was identified, and accordingly, an optimized nanosurface geometry for application as an orthopedic implant was proposed. According to the results of this study, nanoporous stainless steel surfaces with the nanopore diameter of 100 nm can serve as ideal orthopedic implants. The findings of this study may help the biomedical engineers to engineer nanotopographies which improve cell-nanosurface interactions, ultimately leading to optimized geometries for applications as implants. Additionally, applications in the fields of biomedical, tissue engineering, and cancer research are expected.

## Abbreviations

ALP: Alkaline phosphatase
BSA: Bovine serum albumin
C: Carbon
Co2: Carbon dioxide
Cr: Chromium
Cu: Copper
Dapi: 4′,6-diamidino-2-phenylindole
DMEM: Dulbecco modified Eagle’s medium
ECM: Extracellular matrix
EDTA: Ethylene diamine tetraacetic acid
FBS: Fetal bovine serum
FITC: Fluorescein isothiocyanate
IF: Immunofluorescence
Mn: Manganese
Mo: Molybdenum
N: Nitrogen
NaCl: Sodium chloride
Ni: Nickel
OD: Optical density
SEM: Scanning electron microscopy
UV: Ultraviolet
